# Lipopolysaccharide with long O-antigen is crucial for *Salmonella* Enteritidis to evade complement activity and to facilitate bacterial survival in vivo in the *Galleria mellonella* infection model

**DOI:** 10.1007/s00430-024-00790-3

**Published:** 2024-05-20

**Authors:** Eva Krzyżewska-Dudek, Vinaya Dulipati, Katarzyna Kapczyńska, Mateusz Noszka, Carmen Chen, Juha Kotimaa, Marta Książczyk, Bartłomiej Dudek, Gabriela Bugla-Płoskońska, Krzysztof Pawlik, Seppo Meri, Jacek Rybka

**Affiliations:** 1 Department of Immunology of Infectious Diseases, Hirszfeld Institute of Immunology and Experimental Therapy, Polish Academy of Sciences, Wrocław, Poland; 2 Department of Bacteriology and Immunology, Translational Immunology Research Program, University of Helsinki, Helsinki, Finland; 3 Department of Microbiology, Hirszfeld Institute of Immunology and Experimental Therapy, Polish Academy of Sciences, Wrocław, Poland; 4 VTT Technical Research Centre of Finland Ltd, Espoo, Finland; 5 Department of Microbiology, Faculty of Biological Sciences, University of Wrocław, Wrocław, Poland; 6 Platform for Unique Models Application (P.U.M.A), Department of Pharmaceutical Microbiology and Parasitology, Faculty of Pharmacy, Wroclaw Medical University, Wrocław, Poland; 7 HUSLAB Diagnostic Center, Helsinki University Central Hospital, Helsinki, Finland

**Keywords:** O-antigen, Long O-antigen, Very long O-antigen, Complement, *Salmonella* Enteritidis, *Galleria mellonella*

## Abstract

Bacterial resistance to serum is a key virulence factor for the development of systemic infections. The amount of lipopolysaccharide (LPS) and the O-antigen chain length distribution on the outer membrane, predispose *Salmonella* to escape complement-mediated killing. In *Salmonella enterica* serovar Enteritidis (*S*. Enteritidis) a modal distribution of the LPS O-antigen length can be observed. It is characterized by the presence of distinct fractions: low molecular weight LPS, long LPS and very long LPS. In the present work, we investigated the effect of the O-antigen modal length composition of LPS molecules on the surface of *S*. Enteritidis cells on its ability to evade host complement responses. Therefore, we examined systematically, by using specific deletion mutants, roles of different O-antigen fractions in complement evasion. We developed a method to analyze the average LPS lengths and investigated the interaction of the bacteria and isolated LPS molecules with complement components. Additionally, we assessed the aspect of LPS O-antigen chain length distribution in *S*. Enteritidis virulence in vivo in the *Galleria mellonella* infection model. The obtained results of the measurements of the average LPS length confirmed that the method is suitable for measuring the average LPS length in bacterial cells as well as isolated LPS molecules and allows the comparison between strains. In contrast to earlier studies we have used much more precise methodology to assess the LPS molecules average length and modal distribution, also conducted more subtle analysis of complement system activation by lipopolysaccharides of various molecular mass. Data obtained in the complement activation assays clearly demonstrated that *S*. Enteritidis bacteria require LPS with long O-antigen to resist the complement system and to survive in the *G. mellonella* infection model.

**Supplementary Information:**

The online version contains supplementary material available at 10.1007/s00430-024-00790-3.

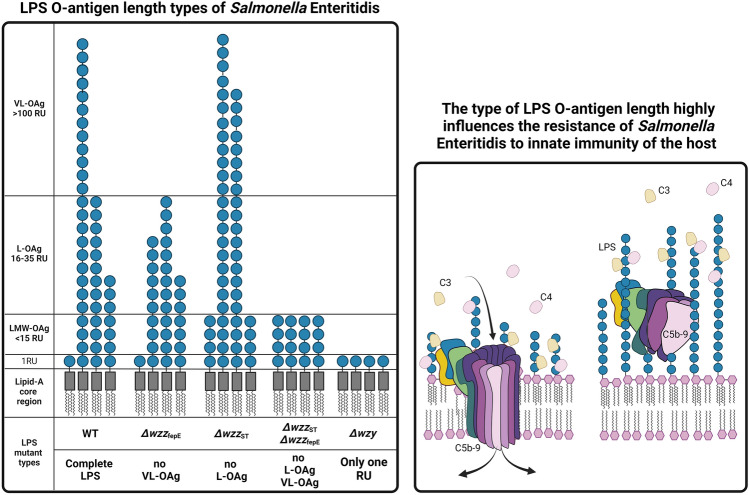

## Introduction

*Salmonella* bacteria are facultative intracellular pathogens of worldwide epidemiological importance with a wide range of hosts [1]. In the genus *Salmonella*, according to 16S rRNA sequence analysis, two species can be distinguished: *Salmonella enterica* (*S. enterica*) and *Salmonella bongori* (*S. bongori*) [2, 3]. *S. enterica* species is further divided into six subspecies: *enterica*, *salamae*, *arizonae*, *diarizonae*, *houtenae* and *indica* on the basis of biochemical and phylogenetic relationship. Based on the presence of antigenic determinants (somatic antigen O, ciliary antigen H) *S. enterica* is further subdivided into serovars. According to the World Health Organization, more than 2600 *Salmonella* serovars are distinguished using the Kauffmann-White classification, and new serovars are still being discovered [2, 3].

*Salmonella* bacteria are among the most commonly isolated gastrointestinal pathogens and the second leading cause of gastrointestinal infection in the European Union (EU). They are mainly transmitted through the fecal–oral route [4]. *Salmonella* bacteria can be classified into typhoidal and non-typhoidal *Salmonella* strains (NTS). *Salmonella* Typhi (*S.* Typhi) strains are the agents that cause typhoid fever, whereas *Salmonella* Paratyphi (*S.* Paratyphi) bacteria of groups A, B and C cause paratyphoid fever. Unlike NTS, infections caused by typhoidal strains occur only in humans. They have high mortality rates mainly in underdeveloped regions [5, 6]. NTS include strains other than *S.* Typhi and *S.* Paratyphi. NTS are the etiological agent of salmonellosis of various clinical forms, of which the gastrointestinal form is the most common. Globally, NTS predominantly cause a self-limiting disease characterized by abdominal cramps, diarrhea and vomiting [7]. In immunocompetent individuals, secondary bacteremia is thought to be uncommon. However, in Sub-Saharan Africa NTS strains can cause invasive infections (bloodstream infections, meningitis) in immunocompromised individuals [8, 9]. Serovars *Salmonella enterica* Enteritidis (*S.* Enteritidis) and *Salmonella enterica* Typhimurium (*S.* Typhimurium) are among the most common NTS isolated from humans in the EU. However, their global distribution is not uniform [10, 11].

Lipopolysaccharide (LPS) is the main component of the outer membrane of Gram-negative bacteria and a key virulence factor of NTS strains [12]. LPS forms a barrier between the cell and the external environment, providing the bacteria resistance to antibiotics and detergents. LPS also enables the survival of bacteria in the host organism during colonization, protecting the cell from complement-dependent cytotoxicity (CDC) and opsonophagocytosis [13]. The inflammatory potential of LPS triggers a strong innate immune response in the host, leading to cell lysis or even septic shock [14]. LPS consists of three main structural parts: lipid A, oligosaccharide core and O-antigen (O-Ag). Lipid A is the most conserved LPS domain, while O-Ag has a very high structural diversity among bacterial strains. The O-Ag chain is built up of oligosaccharide repeating units (RUs), with its saccharide structure differing among various strains of a bacterial species. The structural diversity of O-Ag as the distal part of LPS, contribute to its immunogenic properties [15]. The O-antigen has a variable number of RUs, varying from one to even a hundred in a single strain and in a single bacterial cell [16]. It has been shown that in one LPS preparation there may be molecules with high range of molecular weight, due to a varying number of RUs [17, 18].

In *S*. Enteritidis and *S*. Typhimurium a modal distribution of the O-antigen length can be observed. It is characterized by the presence of distinct fractions: (i) low molecular weight O-Ag LPS (LMW-OAg LPS)—an O-Ag composed of fewer than 15 RUs, (ii) long O-Ag LPS (L-OAg LPS) with 16–35 RUs and (iii) LPS with very long O-Ag LPS (VL-OAg LPS) with more than 100 RUs [19, 20]. The number of RUs attached to the lipid A-core region is regulated by *wzz* genes. The gene *wzz*_ST_ is responsible for the synthesis of L-OAg LPS, while *wzz*_fepE_ controls the VL-OAg LPS synthesis [19, 21–23]. It has been shown that such a modal distribution of LPS length can be found among various *Enterobacteriaceae* [19, 23–26], but VL-OAg LPS often remains unrecognized due to difficulties in its detection.

Numerous studies have shown that the proportions of individual fractions of LPS molecules on the bacterial cell surface are important in the protection from complement killing [19, 27–33]. However, the literature data about that influence is incoherent. Studies on *S*. Typhimurium initially indicated greater importance of the *wzz*_fepE_ gene, and thereby the VL-OAg LPS fraction, in protecting the bacteria from complement killing [29]. However, results obtained in later experiments indicated that the L-OAg LPS (*wzz*_ST_ gene) fraction is essential for bacterial survival in human serum [30, 34]. It should be emphasized that most of the data regarding the involvement of different LPS O-Ag fractions contributing to *Salmonella* complement evasion were collected based on experiments carried out on *S.* Typhimurium serovars. Therefore, the role of VL-OAg and L-OAg LPS in other serovars in bacterial serum resistance is still unclear. Grossman et al. showed that even minor differences in the orientation of –OH groups in the RUs in *S*. Typhimurium and *S*. Enteritidis could lead to differences in the activation levels of complement C3 via the alternative pathway [31]. Furthermore, the studied bacterial strains often lack an in-depth characterization of their LPS O-antigen fractions. Most experiments describing *Salmonella* serum resistance are based on simple serum susceptibility assays, lacking clear evidence of the various modes of bacterial evasion mechanisms to prevent complement killing. In the present work, we investigated the effect of the O-antigen modal length composition of LPS molecules on the surface of *S*. Enteritidis cells on its ability to evade host complement responses. In order to determine the roles of different LPS types in complement evasion we constructed a panel of chromosomal mutants with different O-antigen fractions, developed a method allowing the analysis of the average LPS lengths and investigated the interaction of the bacteria and isolated lipopolysaccharides with complement components. Additionally, we assessed the aspect of LPS O-antigen chain length in *S*. Enteritidis virulence in vivo in the *Galleria mellonella* infection model.

## Materials and methods

### Bacterial strains and culture conditions

Bacterial strains and plasmids used in this study are presented in Table 1. Bacteria were grown in lysogeny broth medium (LB) (Biocorp). Solid medium contained 1.5% (w/v) agar (Biocorp). Ampicillin (100 µg/ml) or kanamycin (50 µg/ml) (Sigma-Aldrich) were added when appropriate.

**Table 1:** Bacterial strains and plasmids used in this study

Strain name	Strain/plasmid characteristic	LPS O-Ag phenotype	Source/references
*Salmonella* Enteritidis (*S*. Enteritidis) PCM 2817 wild-type (WT)	WT	VL-OAg L-OAg LMW-OAg	Polish collection of microorganisms (PCM), HIIET PAS, Wroclaw, Poland
*S.* Enteritidis PCM 2817∆*wzz*_ST_	wzz_ST_::FRT	VL-OAg LMW-OAg	This study
*S.* Enteritidis PCM 2817∆*wzz*_fepE_	wzz_fepE_::FRT	L-OAg LMW-OAg	This study
*S.* Enteritidis PCM 2817∆*wzz*_ST_∆*wzz*_fepE_	wzz_ST_::FRT wzz_fepE_::FRT	LMW-OAg	This study
*S.* Enteritidis PCM 2817∆*wzy*	wzy::FRT	one RU	This study
*Escherichia coli* (*E. coli*) BW25113/pKD46	λ red mutagenesis plasmid	–	Coli Genetic Stock Center; [35]
*E. coli* BW25141/pKD4	Template plasmid for FRT cassette	–	Coli Genetic Stock Center; [35]
*E. coli* BT340/pCP20	Plasmid for thermal induction of FLP recombinase synthesis	–	Coli Genetic Stock Center; [35]

*OAg* O-antigen, *VL* very long, *L* long, *LMW* low molecular weight, *RU* repeating unit, *HIIET PAS* Hirszfeld Institute of Immunology and Experimental Therapy, Polish Academy of Sciences

### Mutagenesis of *S*. Enteritidis LPS biosynthesis genes

Mutagenesis was performed according to the phage λ Red-mediated homologous recombination method described by Datsenko and Wanner [35]. In brief, wild-type *S*. Enteritidis PCM 2817 was first transformed with the Red helper plasmid pKD46 and subsequently transformed with PCR products that were generated using pKD4 as a template plasmid, which contains FRT-flanked kanamycin (*kan*) resistance gene. Linear DNA fragments and competent cells were prepared according to the method described by Czerniak and Hensel [36]. The sequences of the oligonucleotide primers used in this study are available on request. To obtain nonpolar deletion mutants, *kan* resistance genes were removed by transforming the mutants with plasmid pCP20, which encodes the FLP recombinase system [37]. The lack of appropriate genes was verified by PCR analysis after transformation with the Red helper plasmid pKD46, with PCR-products, with the pCP20 plasmid and to confirm the removal of the *kan* resistance genes, and sequencing. Additionally corresponding changes in the modal distribution pattern of LPS were verified by SDS-PAGE and GC–MS analysis of LPS monosaccharide components.

### LPS isolation and analysis by SDS-PAGE

LPS was isolated by the hot phenol–water method [38]. Briefly, freeze-dried bacteria were suspended in 45% phenol solution (2 g/50 ml) and incubated at 65 °C with intermittent stirring for 15 min. The suspension was cooled to 5 °C, centrifuged (3500 *g*, 30 min), and the aqueous phase was collected. Water was added to compensate for the collected aqueous phase volume, and the cycle was repeated. The aqueous phases were combined and dialyzed against deionized water to remove residual phenol, then filtered and freeze-dried. The obtained crude LPS was dissolved in MiliQ water and purified by ultracentrifugation (105,000 *g*, 6 h, 4 °C) [39]. LPS extracts were analyzed by SDS-PAGE using the Laemmli buffer system [40]. Electrophoresis was performed using 6% polyacrylamide stacking gels and 15% separating gels. Samples were loaded into the gels after mixing with Laemmli buffer and heating at 98 °C for 4 min. The SDS-PAGE separation of LPS was performed at constant voltage (120 V), for 90 min using a Mini-Protean Tetra Cell apparatus (Bio-Rad). The separated LPS was visualized using silver staining according to Tsai and Frasch [41] with the modification of Fomsgaard [42] and imaged under white light using a GelDoc XR system (Bio-Rad).

### GC–MS analysis of monosaccharides

The following three-letter abbreviations of monosaccharides are used throughout the publication: Gal—d-galactose; Hep—heptose, d-*glycero*-d-*manno*-heptose; Kdo—3-deoxy-d-*manno*-oct-2-ulosonic acid; Man—d-mannose; MuAc—*N*—acetylmuramic acid; Rha—l-rhamnose, 6-deoxy-l-mannose; Tyv—tyvelose, 3,6-dideoxy-d-*arabino*-hexose.

### Preparation of alditol acetate derivatives of monosaccharides from bacterial mass samples for the analysis of O-antigen monosaccharide composition

To determine the monosaccharide composition of LPS O-antigen, sugar analysis using alditol acetate derivatives was performed. Briefly, bacterial mass (10 mg) was hydrolyzed in 1 ml of 1% CH_3_COOH (Sigma–Aldrich) in water at 100 °C for 30 min, followed by centrifugation (15 min, 12,000*g*). HCl (Sigma-Aldrich) was added to the supernatant to a final concentration of 1 M and hydrolysis was carried out at 80 °C for 2 h. After hydrolysis, the sample was neutralized with NaOH (Merck Millipore). After drying under a stream of N_2_ (Air Products) and rinsing twice with methanol (POCH), the sample was reduced with 1 M NaBH_4_ (Sigma–Aldrich) solution in water at 4 °C for 24 h. After the reduction, NaBH_4_ was decomposed with acetic acid, the sample was dried under a stream of N_2_. After washing twice with methanol the sample was subjected to acetylation: 50 µl of pyridine (Merck Millipore) and 250 µl of acetic anhydride (POCH) were added and incubated at 100 °C for 30 min. Then, the sample was dried under a stream of N_2_, washed twice with methanol and extracted in a chloroform: water system (POCH). The chloroform phase was collected, dried under a stream of N_2_ and dissolved in 100 µl of ethyl acetate (Sigma–Aldrich) for gas chromatography coupled with mass spectrometry (GC–MS) analysis.

### Preparation of acetylated methylglycosides and acetylated methylglycoside methyl esters from LPS and bacterial mass samples

Bacterial mass (10 mg) was hydrolyzed in 4 M HCl in methanol at 80 °C for 17 h. After incubation, the supernatant was evaporated under a stream of N_2_ and washed twice with methanol. The sample was acetylated: 20 µl of pyridine and 100 µl of acetic anhydride were added and the sample was heated at 100 °C for 30 min, dried under a stream of N_2_, washed twice with methanol and extracted in a chloroform: water system. The chloroform phase was collected, dried under a stream of N_2_ and dissolved in 100 µl of ethyl acetate for GC–MS analysis. LPS (1 mg) was hydrolyzed in 0.75 M HCl in methanol at 80 °C for 1 h, followed by neutralization with NaOH in methanol to a slightly alkaline pH. The sample was centrifuged (5 min, 12,000 *g*), and the supernatant was evaporated under a stream of N_2_ and washed twice with methanol. Acetylation and extraction in the chloroform: water system were carried out analogously to the bacterial mass. The resulting acetylated methylglycosides of Tyv and Hep and acetylated methylglycoside methyl esters of Kdo and MuAc were analyzed by GC–MS technique. The experiment was performed in four biological replicates.

### GC–MS analysis

Analysis of the monosaccharide derivatives: acetylated methylglycosides and acetylated methylglycoside methyl esters, was carried out using a FOCUS gas chromatograph (Thermo-Scientific) equipped with Zebron ZB-5HT 30 m × 0.25 mm × 0.25 μm w/5 m guard column (Phenomenex) coupled to ITQ 700 ion trap type mass detector (Thermo-Scientific). The analyzed derivatives were acetylated methylglycosides of Tyv, Rha and Hep or methylglycoside acetylated methyl esters of MuAc and Kdo. For Tyv four derivatives of each monosaccharide (α or β anomers with pyranose or furanose ring) were obtained, for Kdo: two derivatives (α and β anomers) and one derivative for Hep or MuAc. The measured amount of each form was added for each sugar. The amounts of Rha or Tyv, as components of the O-specific polysaccharide, were compared to the amounts of Kdo or Hep, as components of the LPS core. The resulting proportions between the O-specific chain and core oligosaccharide components corresponded to the average lengths of the lipopolysaccharide molecules of a given mutant.

### Serum

Normal human serum (NHS) was pooled from blood samples from a group of 15 healthy adults (7 males; 8 females). All donors provided a written informed consent. The blood was drawn into Vacutainer serum tubes with a clot activator (SKU 367820, BD). The blood was allowed to clot, and the sera were subsequently harvested by centrifugation (10 min, 1200 g), pooled in even contribution from each donor, aliquoted and stored at − 70 °C until used. The required volume of serum was thawed on ice immediately before use and each portion was used only once. Heat-inactivated serum (HIS) was generated by incubating NHS for 1 h at 56 °C.

### Serum susceptibility assays

NHS and HIS were distributed onto a Honeycomb 2 plate (Growth Curves AB Ltd) and incubated with or without Ravulizumab (100 µg/ml; Alexion), a humanized monoclonal complement inhibitor (anti-C5) at ambient temperature for 35 min. The investigated *S*. Enteritidis PCM 2817 strains were cultured overnight at 37 °C in 3 ml LB medium. After overnight incubation, bacterial cells were transferred into 5 ml of fresh LB medium and incubated at 37 ℃ with rotation to achieve an OD_600_ value of 0.3 (early exponential phase). Next, bacterial cells were further diluted in 10 mM Tris-HCl buffer (pH = 7.3) supplemented with 150 mM NaCl, 2 mM CaCl_2_ and 0.5 mM MgCl_2_ (Sigma-Aldrich). Bacterial cells were added to the plate (CFU = 10^6^/well). The final serum concentration after addition of bacterial cells was 25%. The plate was incubated for 15 h in a BioScreen C (Growth Curves AB Ltd) incubator at 37 °C with continuous shaking. The OD_600_ of each well was measured every 20 min. The experiment was performed twice with three biological replicates.

### C3 cleavage in serum

The investigated *S*. Enteritidis PCM 2817 strains were cultured overnight at 37 °C in 3 ml LB medium. After overnight incubation, bacterial cells in the early exponential phase were transferred into 5 ml of fresh LB medium and incubated at 37 °C with rotation to achieve an OD_600_ value of 0.3. Next, bacterial cells were collected by centrifugation (10 min, 5000 *g*, 4 °C) and washed three times in phosphate-buffered saline (PBS; 137 mM NaCl, 2.7 mM KCl, 8 mM Na_2_HPO_4_, and 2 mM KH_2_PO_4_ (Sigma–Aldrich), pH 7.4). Bacterial cells (2 × 10^8^ CFU/ml) were incubated in 50% NHS for 10 min at 37 °C with shaking. After incubation the samples were pelleted (10 min, 5000 *g*, 4 °C) and the supernatants were mixed with Bolt^™^ LDS buffer and Bolt^™^ sample reducing agent (Life Technologies), and heated at 95 °C for 5 min. Gel electrophoresis was performed using Bolt^™^ 4–12% Bis–Tris Plus Mini Gels (Invitrogen) on a Mini Gel Tank in Bolt^™^ MES buffer (Invitrogen) at 165 V for 45 min. Western blot analysis was performed using the iBlot 2^™^ system according to the manufacturer’s instructions (Invitrogen). Membrane blocking was carried out with non-fat dried milk in PBS/0.05% Tween 20 (w/v). The products of C3 cleavage by bacteria were detected using primary antibodies: rabbit anti-human C3c (Dako) and secondary antibodies: goat anti-rabbit IgG conjugated with horseradish peroxidase (HRP) (minimal cross-reaction to mouse, human, rat serum proteins, Jackson ImmunoResearch laboratories). After washing, the membrane was incubated in the enhanced chemiluminescence (ECL) substrate containing 1 M Tris HCl, ph 8.5; 250 mM luminol; 90 mM coumaric acid and 30% H_2_O_2_. Enhanced chemiluminescence was detected on Super-RX films (Fujifilm Corporation). The experiment was performed in three biological replicates.

### Functional complement activation assay

Complement activation via the alternative pathway was measured by an enzyme-linked immunosorbent assay based on Seelen et al. [43]. Briefly, a Nunc MaxiSorp plate (Thermo Scientific) was coated overnight with 1 µg/ml of LPS isolated from the WT and *S.* Enteritidis PCM 2817 mutants in PBS containing 10 mM MgCl_2_ (Sigma–Aldrich). The plate was washed three times with PBS containing 0.05% Tween-20 (PBS-T). NHS dilutions were selected on the basis of preliminary tests and prepared in Tris-buffered saline (TBS; 10 mM Tris base and 150 mM NaCl (Sigma–Aldrich), pH 7.4) containing 5 mM MgCl_2_, 10 mM EGTA (MgEGTA, Sigma–Aldrich), 1% w/v bovine serum albumin (BSA), 0.05% Tween-20 and incubated for 1 h at 37 °C. The plate was washed three times with PBS-T. Next, biotinylated anti-human C5b-9 neoepitope antibody (Hycult, clone aE11) in TBS containing 1% w/v BSA (B-TBS) was added to the wells and the plate was incubated for 1 h at 37 ℃. After washing, streptavidin conjugated with horseradish peroxidase (Pierce^™^ Streptavidin Poly-HRP, Thermo Scientific) in B-TBS was added and incubated for 1 h at 37 °C. After washing, the reaction was revealed with 3,3′,5,5′-tetramethylbenzidine (TMB) substrate, stopped with 0.5 M sulphuric acid, and absorbance read at 450 nm. Absorbance readings were normalized to a wild-type value of 100%. The experiment was performed in three biological replicates.

### Detection of C5b-9 deposition on bacterial surfaces

The deposition of C5b-9 on *S*. Enteritidis strains was assessed using flow cytometry. Bacterial cells (CFU/ml = 10^6^) were incubated with 50% NHS or HIS for 30 min at 37 °C and washed three times with PBS. Cells were incubated with the monoclonal mouse anti-human C5b-9 antibody (clone aE11) (Hycult) for 45 min at ambient temperature, washed and detected with an anti-mouse Alexa 488 antibody (Abcam) (45 min, ambient temperature). To assess cell permeability, bacterial cells were stained with 7AAD (Invitrogen) according to the manufacturer’s specifications. 7AAD is a fluorescent DNA binding dye, that selectively stains cells with compromised membrane integrity, prior to analysis. Samples were analyzed using the CytoFLEX Flow Cytometry Platform (Beckman Coulter). The experiment was performed in three biological replicates.

### *Galleria mellonella* infection model

*G. mellonella* larvae were obtained from ZooFaktor (Piastów, Poland). Only larvae characterized by a body length of 20 mm (± 5 mm), body weight of 190 mg (± 30 mg), high mobility and a cream color without dark discoloration were used for the experiment. Tested bacterial strains were grown overnight, and washed three times with PBS and diluted to obtain a bacterial inoculum of 10^6^ and 10^7^ CFU/ml. The survival assay was carried out on an experimental group of 15 larvae. Each larvae was injected with 10 μl of bacterial suspension into the hindmost left proleg using a 50 µl syringe (Hamilton, Bonaduz, Switzerland). After injection, the larvae were placed in a 100 mm Petri dish and incubated at 37 °C. PBS-injected larvae (n = 15) were included in the experiment as control. Larval survival was followed for 120 h. The larvae were considered dead when they did not move in response to physical contact, had lost turgor and melanization was visible. The results are expressed as percent of survival. The experiment was performed in four biological replicates.

### Statistical analysis

Calculations were performed using GraphPad Prism (Version 9.2.0). The Shapiro–Wilk test assessed the normality distribution. Ordinary one-way ANOVA with post hoc Dunnett’s or Šídák's analysis were applied to compare groups. The differences with a significance level of p < 0.05 were considered significant.

## Results

### Mutagenesis of *Salmonella* Enteritidis LPS O-antigen length mutants

**Fig. 1:**
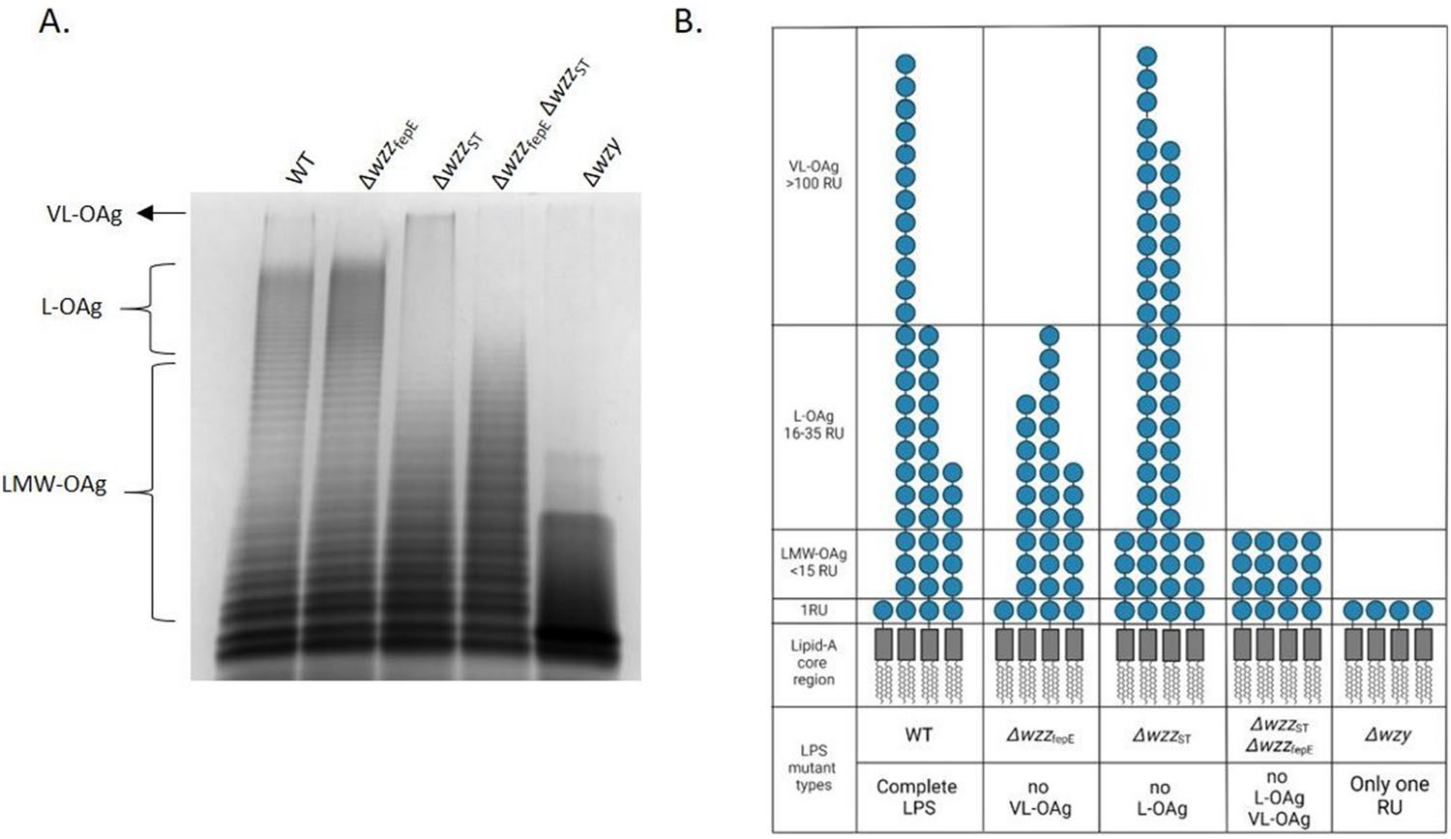
LPS O-antigen length types of *S*. Enteritidis. **A** SDS-PAGE analysis of LPS isolated from *S*. Enteritidis PCM 2817 WT and O-antigen mutants, **B** Schematic overview of the LPS O-antigen length types in *S*. Enteritidis, adapted from [20]

To investigate the role of LPS O-antigen types in complement evasion mechanisms of *Salmonella* Enteritidis a panel of chromosomal mutants of *S.* Enteritidis PCM 2817 differing in the LPS O-antigen modal pattern distribution was constructed. LPS preparations isolated from the WT strain and mutants (Δ*wzz*_fepE_, Δ*wzz*_ST_, Δ*wzz*_ST_Δ*wzz*_fepE_, Δ*wzy*) were analyzed by SDS-PAGE and silver staining (Fig. 1). In comparison with the WT strain, LPS isolated from the Δ*wzz*_fepE_ mutant lacked VL-OAg (Fig. 1) and the Δ*wzz*_ST_ mutant lacked L-OAg (Fig. 1). The LPS of Δ*wzz*_ST_Δ*wzz*_fepE_ lacked VL-OAg and L-OAg while the Δ*wzy* null mutant showed only one O-antigen RU in the LPS molecule (Fig. 1). Interestingly, deletion of the *wzz*_fepE_ gene resulted in a distinct difference in the average L-OAg LPS molecular weight in that strain in comparison to the WT strain (Fig. 1). Additionally, an increased intensity of the VL-OAg LPS bands was observed in the Δ*wzz*_ST_ mutant in comparison to the WT strain (Fig. 1).

### Analysis of the average LPS O-antigen chain length

**Fig. 2:**
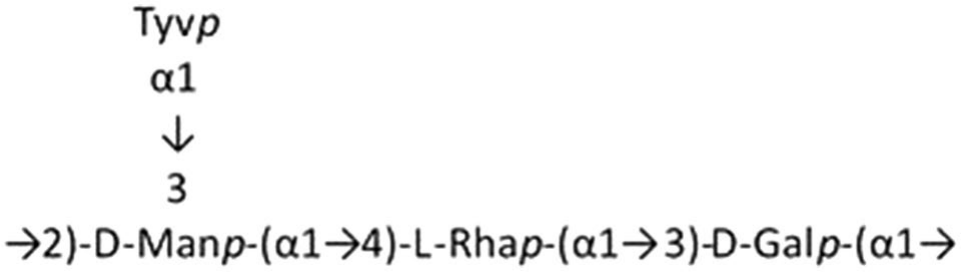
Structure of the O-antigen subunit of *S*. Enteritidis. *Tyv* tyvelose, *Gal* galactose, *Rha* rhamnose, *Man* mannose

The O-antigen subunit of *S.* Enteritidis is a tetrasaccharide containing: Rha, Man, Gal and Tyv (Fig. 2) [44]. The analysis of the average length of O-Ag polysaccharide chain was based on the analysis of the ratio between the components of the O-specific part (Rha or Tyv) and the core part (Kdo or Hep), which allows for the determination of the average LPS length. In addition, the content of MuAc in bacterial cells was measured. The Hep/MuAc ratio (LPS core and peptidoglycan markers respectively) provides the information on relative amounts of LPS molecules on the surface assuming that the amount of mureine in the cell wall is comparatively stable between tested strains. The sugar composition of the LPS molecule was confirmed by alditol acetate sugar analysis using GC–MS. We confirmed the presence of monosaccharides in the sample: Rha and Tyv (components of the LPS O-antigen), and Hep (a component of the LPS core oligosaccharide), which were used as chemical markers for estimation of the average lengths of the LPS O-antigens. Analysis of the average lengths of the O-specific parts of LPS was performed on entire bacterial cells and on LPS preparations isolated from the tested *S.* Enteritidis PCM 2817 strains. Results were expressed as percentage (%), taking the value of 100% for the Δ*wzy* mutant. An example chromatogram, structures of derivatives and mass spectra are presented in the supplementary data, Figs. S1, S2. Analysis of the proportions of Tyv and Rha in the bacterial mass and in the isolated LPS preparations, as well as the proportions of Hep and Kdo in the bacterial mass, showed that there were no significant differences in the ratios obtained from the analysis of the O-antigen and the LPS core markers for the tested *S.* Enteritidis PCM 2817 strains (supplementary data, Fig. S3). The results confirmed that the selected chemical markers of the O-antigen and core parts of the LPS are constant, and that the markers can be used to analyze the average lengths of LPS in the studied strains.

### The average length of LPS O-antigen differs between mutants but the number of LPS molecules is constant

**Fig. 3:**
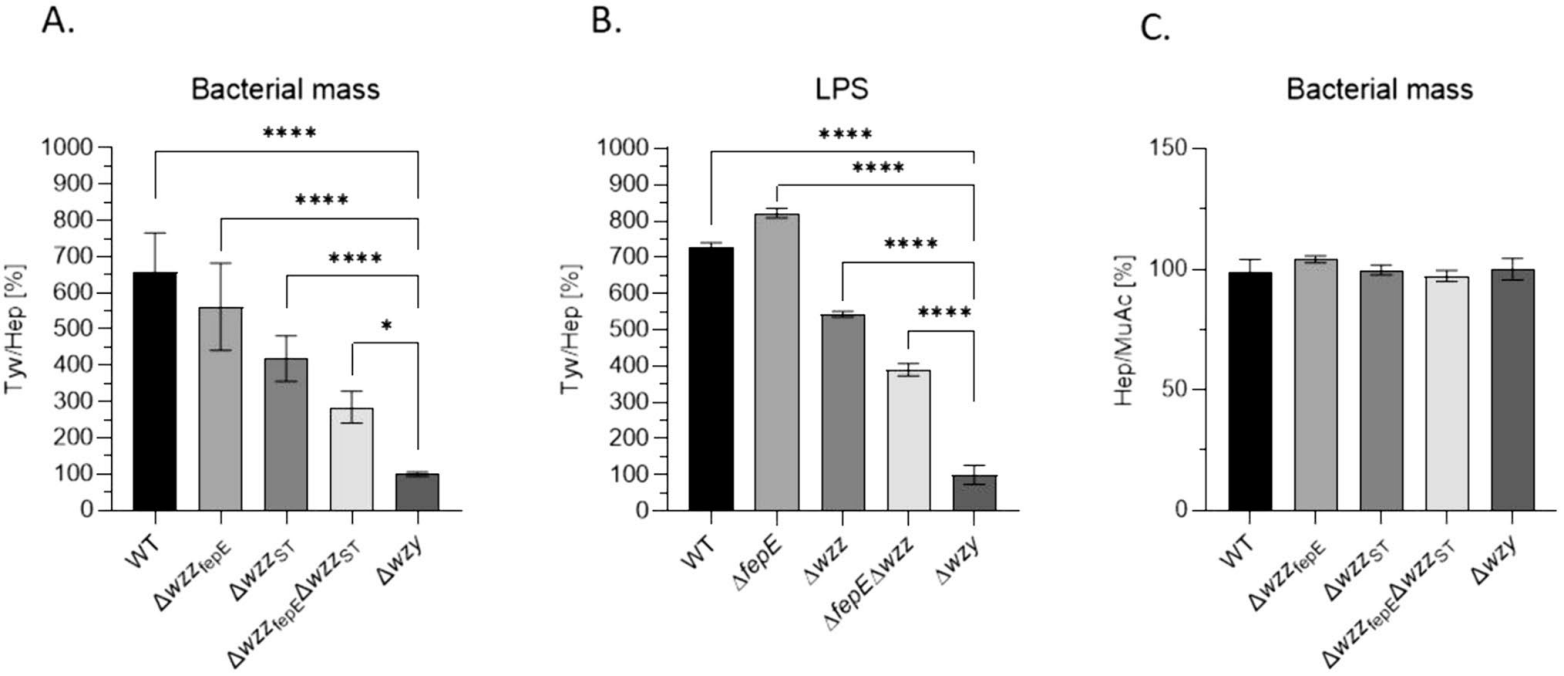
GC–MS analysis of Tyv and Hep content in **A** bacterial cells, **B** isolated LPS preparations together with **C** analysis of Hep and MuAc content in bacterial cells of *S*. Enteritidis PCM 2817 WT and mutant strains. Results are presented as Tyv/Hep or Hep/MuAc expressed as a percentage, taking the value of 100% for the Δ*wzy *mutant. The experiment was performed in four biological experiments. Results were analyzed with ordinary one-way ANOVA and Dunnett's post hoc test (*p < 0.05, **p < 0.01, ***p < 0.001, ****p < 0.0001) assuming Δ*wzy* as the control group. Average from four measurements ± SD

By the analysis of the Tyv/Hep ratio, the average number of repetitive O-antigen subunits per LPS core, i.e. average lengths of the O-specific parts of LPS molecules of the particular *S.* Enteritidis PCM 2817 mutants were determined. The analysis of bacterial mass showed that the LPS of the WT strain had the highest number of O-specific subunits (Tyv/Hep value of 657%), followed by the decrease of the Tyv/Hep ratios for the tested mutants (Fig. 3A). However, the results obtained for isolated LPS preparations were different: the Δ*wzz*_fepE_ mutant had the highest Tyv/Hep ratio (821%), while 726% was obtained for the WT strain, 543% for the Δ*wzz*_ST_ strain and 389% for the Δ*wzz*_ST_ Δ*wzz*_fepE_ strain when compared to Δ*wzy* (100%) (Fig. 3B). Additionally, a comparison of the proportions of the obtained levels of Hep compared to MuAc, found in the bacterial cell wall, indicated a similar number of LPS molecules in the tested strains, as the Hep/MuAc ratio values were similar among strains and ranging from 97 to 104% (Fig. 3C).

### Long O-antigen protects *S*. Enteritidis from complement killing

**Fig. 4:**
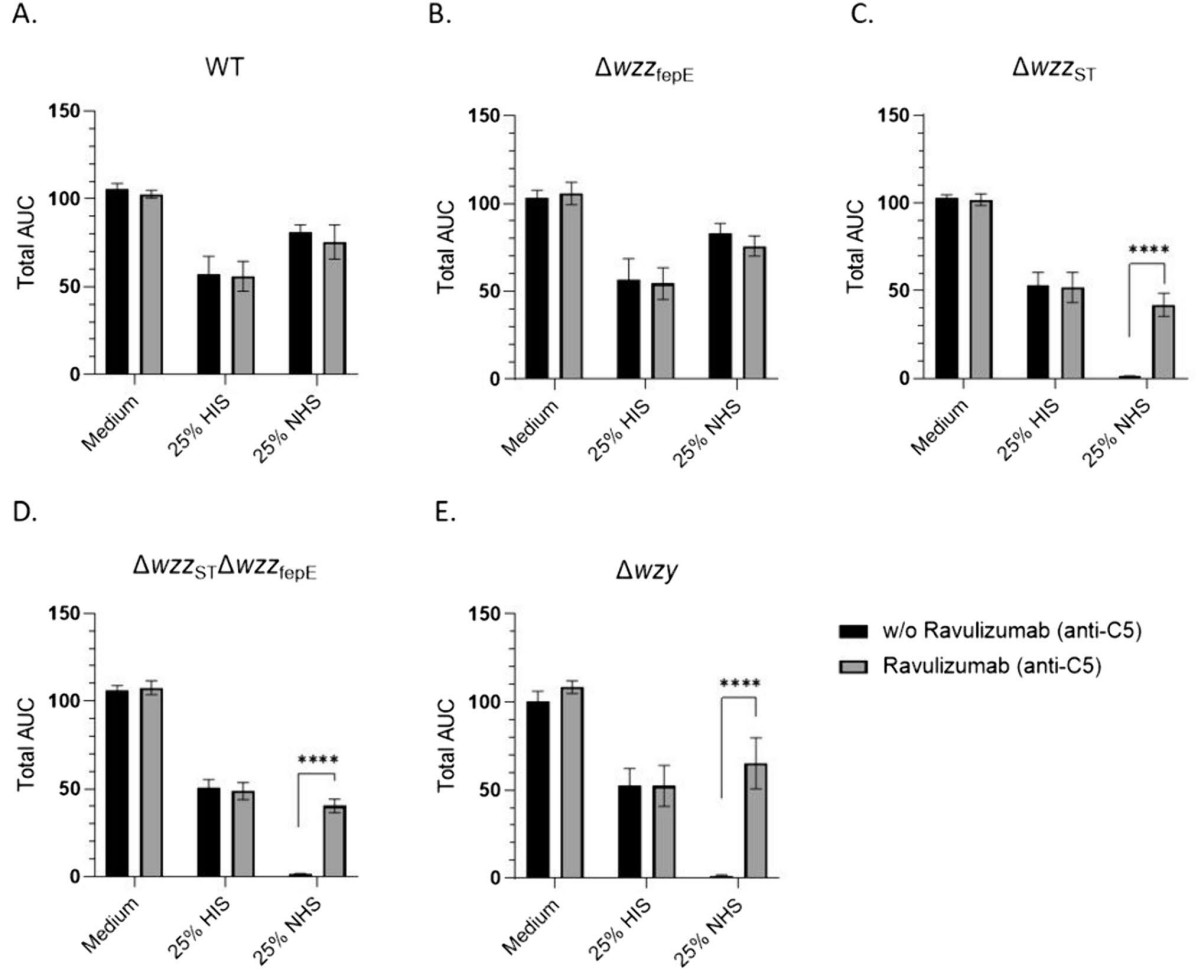
Growth of *S*. Enteritidis PCM 2817 WT (**A**) and mutant strains (**B**–**E**) in medium, 25% HIS and 25% NHS. Results are presented as area under the curve (total AUC). The experiment was performed in six biological replicates. Results were analyzed with ordinary one-way ANOVA and Šídák's post hoc test (*p < 0.05, **p < 0.01, ***p < 0.001, ****p < 0.0001). Average from six measurements ± SD

O-antigen length regulation by *wzz* genes is required for serum resistance in *S.* Typhimurium [19, 29]. To determine the effect of O-antigen length on the survival of *S*. Enteritidis in serum, the tested strains were incubated for 15 h in 25% NHS and OD_600_ values were measured every 20 min. The strains differed in the levels of susceptibility to the bactericidal effect of 25% NHS (Fig. 4). The WT strain and the Δ*wzz*_fepE_ mutant survived in serum after 15 h of incubation (Fig. 4A, B). In comparison, the Δ*wzz*_ST_ mutant, lacking L-OAg in the LPS was killed by serum complement (Fig. 4C). The same pattern of serum sensitivity was observed for the mutants, characterized by the shortest O-antigens: Δ*wzz*_ST_Δ*wzz*_fepE_ and Δ*wzy* (Fig. 4D, E)*.* None of the tested strains were sensitive in the tested conditions to human serum after adding Ravulizumab, a humanized monoclonal complement inhibitor, which inhibits C5 activation and the downstream formation of lytic C5b-9 complex on the cell surface. This indicates a direct involvement of the complement system in killing of the tested *S*. Enteritidis PCM 2817 mutants. These data indicates that L-OAg LPS plays a major role for *S*. Enteritidis in the evasion of the complement system.

### O-antigen length influences complement activation

**Fig. 5:**
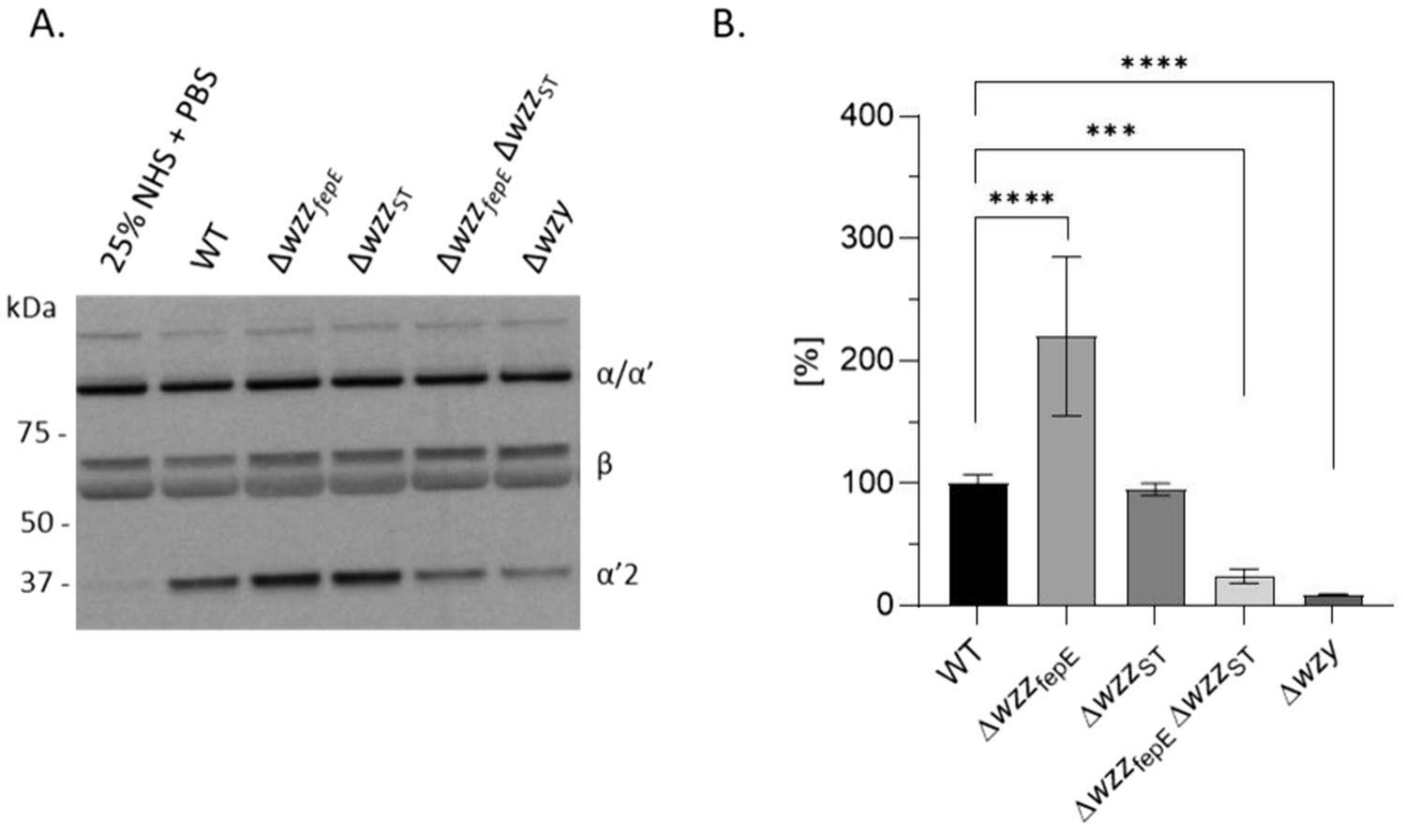
Complement activation of *S*. Enteritidis PCM 2817 WT and mutant strains. **A** Immunoblot of C3 cleavage in 50% NHS. **B** Alternative pathway activation by LPS preparations isolated from the tested *S*. Enteritidis PCM 2817 strains. Activation was measured by ELISA. Experiments were performed in three biological replicates. Data was analyzed by ordinary one-way ANOVA and Dunnett’s post hoc test (*p < 0.05, **p < 0.01, ***p < 0.001, ****p < 0.0001), assuming WT as the control group (100%). Average from six measurements ± SD

In order to test the effect of O-Ag LPS length on C3 activation in serum, the tested *S*. Enteritidis PCM 2817 strains were incubated with 50% NHS and the supernatants were analyzed with a Western blotting technique to detect C3 cleavage. All tested strains activated C3 in serum (Fig. 5A). Upon activation of the complement system, the C3α chain was cleaved into two fragments of approximately 68 (α′) and 39 kDa (α′2). They are the results of C3 activation to C3b and subsequently to inactive iC3b. Comparing to the WT strain the highest amount of the α′2 product was observed for the Δ*wzz*_fepE_ and Δ*wzz*_ST_ mutants, while for Δ*wzz*_ST_Δ*wzz*_fepE_ and Δ*wzy* that amount was much lower. Additionally, the level of complement activation via the alternative pathway by the O-antigen LPS types, isolated from the tested *S.* Enteritidis PCM 2817 strains, was tested by ELISA, where the deposition of C5b-9 on the plate was measured. The level of alternative pathway activation by different LPS types is shown in Fig. 5B. While LPS isolated from the WT strain and the ∆*wzz*_ST_ mutant had similar activation potential, LPS from ∆*wzz*_fepE_ mutant activated the alternative complement system twice as effectively as the wild-type strain (220% for ∆*wzz*_fepE_ versus WT). Upon shortening of the average LPS length, the level of complement activation decreased: ∆*wzz*_fepE_∆*wzz*_ST_ mutant activated complement by 24%, while the ∆*wzy* mutant only by 9% (Fig. 5B). The obtained data suggests that VL-OAg LPS modulates activity of the alternative pathway, as the activation potential of LPS from ∆*wzz*_fepE_ mutant differs distinctly from that of the ∆*wzz*_ST_ and WT strains.

### Long O-antigen is required for protection from serum complement

**Fig. 6:**
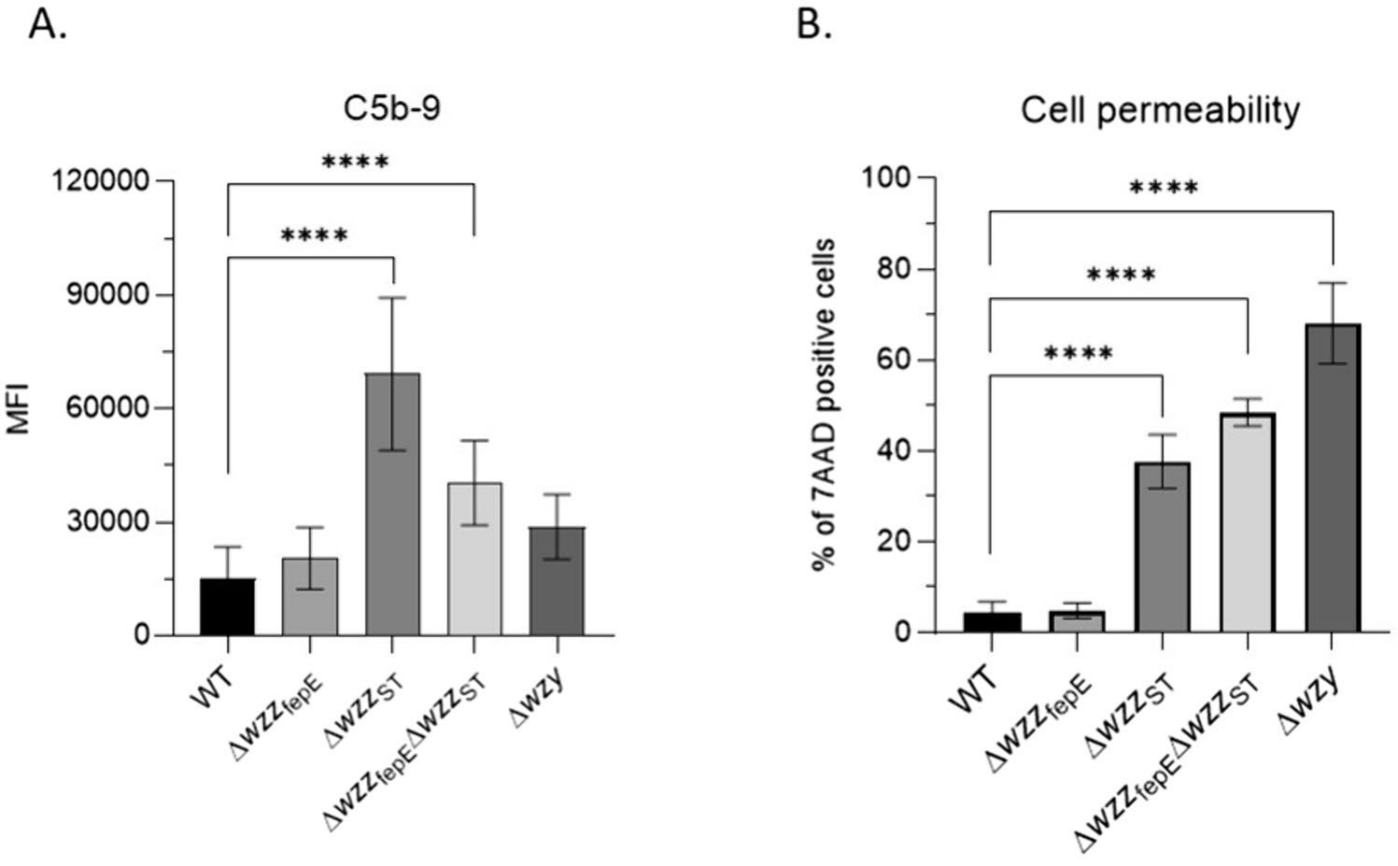
C5b-9 deposition and the effect of complement attack on the cell membrane integrity of *S*. Enteritidis PCM 2817 WT and mutant strains. **A** Mean fluorescence intensity (MFI) of C5b-9 neo-epitope deposition on bacterial cells. **B** Frequency of 7AAD positive cells; the percentage of 7AAD positive cells stands for the percentage of cells with compromised membrane integrity. The experiment was performed in three biological replicates. Data was analyzed by ordinary one-way ANOVA and Dunnett's post hoc test (*p < 0.05, **p < 0.01, ***p < 0.001, ****p < 0.0001), assuming WT as the control group. Average from six measurements ± SD

After the experiment with isolated LPS we next analyzed C5b-9 binding to whole cells of the *S*. Enteritidis PCM 2817 strains and the effect of complement on outer membrane permeability by flow cytometry. Wild-type and mutant strains were incubated in 50% NHS for 30 min. After washing the deposition of C5b-9 was detected with an antibody recognizing a neo-epitope in the C5b-9 complex. Cell permeability was monitored with the fluorescent DNA binding dye 7AAD, which selectively stains cells with compromised membrane integrity. We observed C5b-9 deposition on all tested strains, but its levels were not equal. The strongest binding was observed for the Δ*wzz*_ST_ mutant followed by the Δ*wzz*_ST_Δ*wzz*_fepE_ and Δ*wzy* mutants. The level of C5b-9 deposition for the WT strain and Δ*wzz*_fepE_ mutant was only about one third of that of the Δ*wzz*_ST_ mutant (Fig. 6A). No C5b-9 was detected on bacteria incubated with HIS (data not shown). Cells with permeabilized outer membranes were detected for all tested strains. However, their amounts were more abundant for the strain lacking L-OAg (Δ*wzz*_ST_) in comparison to the WT strain and Δ*wzz*_fepE_ mutant (Fig. 6A, B).

### Long O-antigen is required for full virulence of *S*. Enteritidis in the *G. mellonella* infection model

**Fig. 7:**
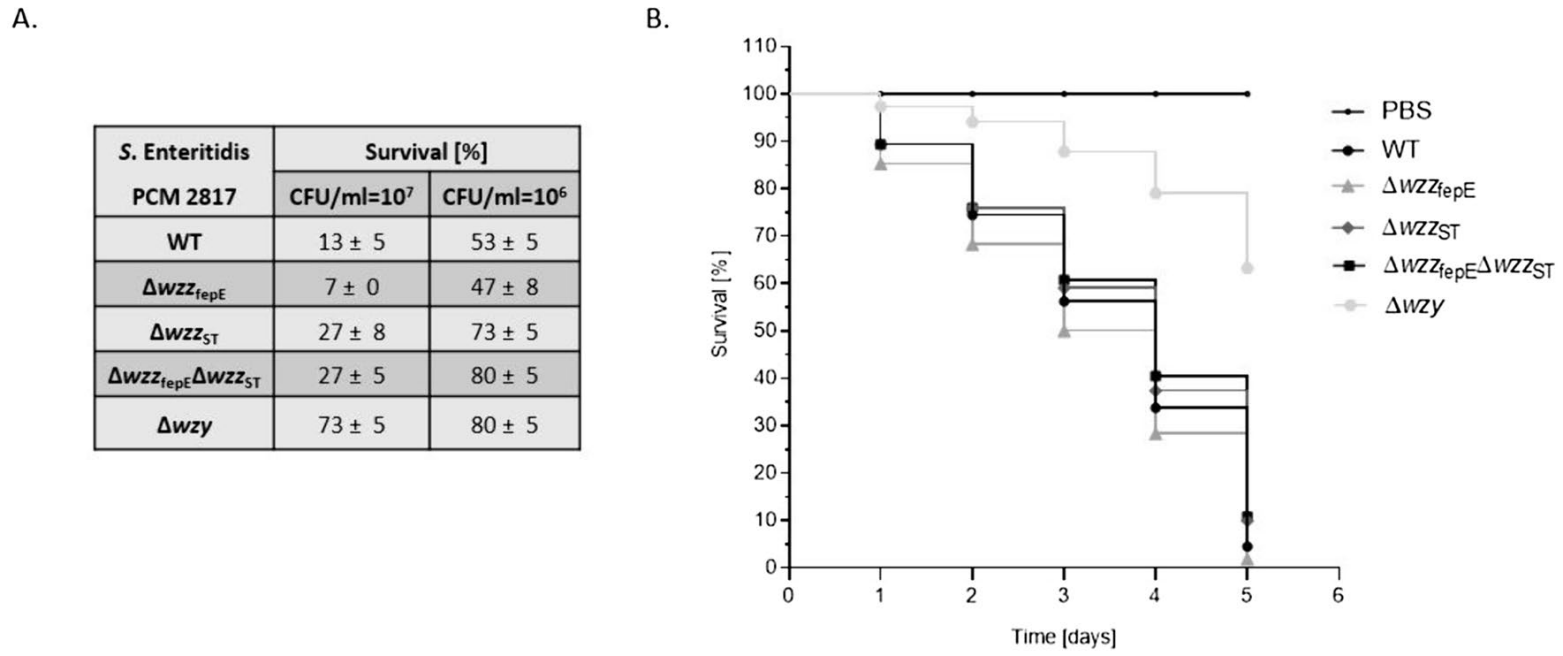
Survival of *G. mellonella* larvae after challenge with *S*. Enteritidis PCM 2817 WT and mutant strains. **A** Survival percentage of larvae after 5 days incubation, **B** Survival curve of larvae over time after injection with 10^7^ CFU/ml. The experiment was performed in four biological replicates. Average from four measurements ± SD

In order to determine the influence of the O-antigen chain length on the pathogenic potential of *S*. Enteritidis, we studied the susceptibility of *G. mellonella* larvae to infection with the wild-type strain and LPS O-antigen mutants. *G. melonella* larvae were challenged with 10^6^ and 10^7^ CFU/ml bacteria, incubated at 37 °C and monitored for signs of melanization for 120 h. Melanization is a part of the insects’ immune response and is used as a measure to determine the insects’ health status after a challenge with pathogens. The survival rate of larvae after 120 h challenge is shown in Fig. 7A. Infection with WT *S*. Enteritidis PCM 2817 strain resulted in a high level of pathogenicity in vivo. After 120 h of incubation, the survival rates of larvae were 13% and 53%, respectively for 10^7^ and 10^6^ CFU/ml infection inoculum. The elimination of VL-OAg LPS (∆*wzz*_fepE_ mutant) reduced the survival of *G. mellonella* larvae to 7% and 47%, respectively, for both amounts of bacteria after 120 h. However, the survival rate of *G. mellonella* increased after challenge with the ∆*wzz*_ST_ mutant (27% and 73% for 10^7^ and 10^6^ CFU/ml infection inocula, respectively). This showed that an intact LPS with L-OAg is required to resist the innate immune response of *G. mellonella* larvae. Infection of larvae with the double knock-out mutant ∆*wzz*_ST_∆*wzz*_fepE_ resulted in a similar survival level of *G*. *mellonella* as in the two tested conditions. Infection with the ∆*wzy* mutant resulted in an increase of *G. mellonella* survival to 73% after injection of 10^7^ CFU/ml bacteria (Fig. 7A). The survival of *G. mellonella* over time is presented in Fig. 7B. In the experiment, a group of 15 larvae injected with PBS buffer served as a control. After 120 h of incubation, larval survival in the control group was 100% (Fig. 7B).

## Discussion

*Salmonella* bacteria are zoonotic pathogens of major importance to human and animal health worldwide. According to the European Food Safety Authority and the European Center for Disease Prevention and Control, salmonellosis remains the second most frequently reported gastrointestinal infection in humans after campylobacteriosis. In 2018, *Salmonella* bacteria were responsible for nearly a third of foodborne disease outbreaks in the EU. Slovakia, Spain and Poland were responsible for 67% of all salmonellosis outbreaks in the EU [45]. *Salmonella* bacteria are characterized by several virulence factors. An infection can lead, apart from diarrhea, to extraintestinal infections and sepsis.

One of the main features that allows *S.* Enteritidis bacteria to survive in an extremely unfavorable environment is LPS. Literature data about the involvement of different LPS O-antigen types in complement evasion by *S*. Typhimurium is ambiguous [29, 30, 34]. Therefore, we examined systematically, using specific deletion mutants, roles of VL-OAg, L-OAg and LMW-OAg LPS in complement evasion by *S*. Enteritidis. First, we characterized the structural properties of the LPS molecules. The measurement of LPS length and its density on the bacterial surface is difficult due to various reasons. Lipopolysaccharides have high structural variability, several LPS length types exist simultaneously in one bacterial cell, and LPS molecules have a tendency to form aggregates of different sizes and experimentally obtained LPS preparations are heterogeneous.

In order to characterize the average LPS O-antigen length of *S*. Enteritidis PCM 2817 WT strain and mutants (Fig. 1) a method measuring the average length of LPS molecules present in a given bacterial strain was used (Figs. 3 and S3). The essence of the used method is the analysis of the ratio between the amount of sugar components present in the RUs of the O-specific part (length-dependent component) and in one of the LPS core components (length-independent component). This approach was previously used for *E. coli* O111 LPS, where the colitose to Kdo ratio was measured colorimetrically [17]. To measure the NeuAc(sialic acid) to Kdo ratio in LPS of *Salmonella* O48 bacteria GC–MS technique was used [33, 46]. In this study, we determined the amount of the chemical markers of the O-specific part of LPS: rhamnose and tyvelose (Fig. S3A, B), as well as of the components of the LPS core: Kdo and heptose (Fig. S3C) using the peracetylated methylglycoside derivatives for the analysis [47]. Rhamnose is a common component of the O antigen [48, 49] and fimbriae [50, 51]. In turn, tyvelose occurs only in some serovars of *Yersinia pseudotuberculosis* [52], *Y. entomophaga* [53], *S*. Enteritidis and *S*. Typhi [48]. Tyvelose is also a component of the immunogenic glycoprotein TSL-1 from *Trichinella spiralis* [54]. The low prevalence of tyvelose as a structural component in bacteria and nematodes makes it an interesting chemical marker for the rapid detection of *S*. Enteritidis. The method used for the average LPS O-Ag chain length analysis is based on the simultaneous analysis of five sugar markers in one sample: Rha, Tyv, Hep, Kdo and MuAc. This method represents a significant advance over the previously used and described method engaging sialic acid as a marker of LPS length, due to the fact that when *Salmonella* does not produce NeuAc in the O-antigen, such a method cannot be used [55].

The obtained results of the measurements of the average LPS length (Fig. 3A, B) confirmed that they can be used for comparison between strains. Differences in the results obtained from the measurement of the proportions of sugar markers in the whole bacterial mass (Fig. 3A) and in the isolated LPS preparations (Fig. 3B) for the WT strain and the Δ*wzz*_fepE_ mutant may be due to the used LPS isolation method. The LPS preparations used for the measurements were isolated using the hot phenol–water method, which is recommended for the isolation of very long and long LPS types [38, 56]. The inclusion in the analysis of muraminic acid also allows to compare the proportion of the total numbers of LPS molecules in the cells between individual strains, assuming that the amount of muramine in the cell is constant (Fig. 3C). The analyzed markers differ significantly in their chemical properties: sensitivity to degradation and susceptibility to disruption of the bonds formed in the LPS molecule. Tyv and Kdo are readily released during the methanolysis reaction. However, their methyl glycosides are sensitive to acid degradation, while efficient release of MuAc from peptidoglycan requires longer methanolysis at a higher concentration of HCl. In addition, ester groups in MuAc and Kdo derivatives are sensitive to elevated pH, which hampers the repetitive neutralization of the mixture after the reaction. The method developed may in the future be the basis for a more detailed analysis of the proportions of different types of LPS (VL-OAg, L-OAg, LMW-OAg) on the bacterial cell surface.

In our previous publication we analyzed 21 *Salmonella* O48 clinical isolates and showed a high variability in the average length of the O-antigen part of LPS molecules ranging up to two orders of magnitude between extreme values [46]. Subsequent experiments on selected strains have shown that even in the same strain the average length can be gradually increased by the bacteria after stimulation with human serum [33]. The mechanisms for the regulation of O-specific chain length during LPS synthesis are still unclear and there are several hypotheses regarding this phenomenon, involving the regulation by synthesis time (the “molecular clock”) [22], or by the length of emerging O-polysaccharide chain (“molecular ruler”) [57]. Recent studies indicate, that the regulation is effected by the expression ratio between the enzymes: WbdA responsible for the elongation of polysaccharide and WbdD responsible for the termination of the synthesis process [58]. A more detailed description of length control of the O-antigen by Wzz proteins was described previously [20]. Additionally, the effect of environmental factors on length regulation is so far very poorly understood and limited to studies on the effect of bactericidal components of the complement system, composition of medium, temperature and also growth phase [31, 34, 59, 60].

Current knowledge in this area is ambiguous. Palva and Mäkäla showed that the LPS on the surface of *S*. Typhimurium has a low content of molecules with 2–18 repeating O-specific subunits (LMW-OAg) and a more predominant LPS containing 19–34 repeating subunits (L-OAg) (77%) [61]. It is worth noting that, at the time of publication of the study, the form of VL-OAg LPS and the *wzz*_fepE_ gene responsible for its formation were not yet known. Despite the presence of bands in the electropherograms indicating very long O-specific chains, the authors do not mention the possibility of the occurrence of LPS with more than 100 repeating O-specific subunits [61]. Numerous studies have shown that the proportions of individual length forms of LPS molecules on the surface of the bacterial cell have a significant impact on the protection of *Salmonella* against the complement system [19, 29, 30, 34, 62]. Previous studies on this phenomenon were performed using mostly *S.* Typhimurium. That serotype, although closely related to *S.* Enteritidis, differ in the composition of its O-antigen structure: in *S*. Typhimurium the monosaccharide tyvelose, present in *S.* Enteritidis in the repeating unit, is replaced by abequose. It has been shown previously that seemingly such a small difference as the orientation of the −OH groups in the positions C2 and C4 in one monosaccharide of the O-antigen repeating unit has such a distinct impact on the opsonization capacity of those serovars [63].

Therefore, in this the study, the influence of different *S*. Enteritidis O-antigen LPS types (Figs. 1, 3) on complement evasion was determined. The analysis of survival in 25% NHS showed that the wild-type strain and Δ*wzz*_fepE_ mutant were serum resistant (Fig. 4A, B), while Δ*wzz*_ST,_Δ*wzz*_fepE_ Δ*wzz*_ST_ and Δ*wzy* were serum sensitive (Fig. 4C–E). The obtained results for the WT strain are in accordance with a previously published study, where the strain showed resistance to 50% NHS [62]. The addition of Ravulizumab (anti-C5 antibody blocking the terminal pathway of complement) in the serum challenge resulted in the survival of the serum sensitive strains (Fig. 4A–E). This proved the involvement of the complement membrane attack complex in the killing of the tested bacteria. The obtained results indicate a small contribution of VL-OAg in the protection of *S*. Enteritidis bacteria against the lytic effect of the complement system. In contrast, L-OAg clearly protects the bacterial cells from complement mediated lysis.

Literature data initially indicated a greater involvement of the *wzz*_fepE_ gene, and thus VL-OAg, in protection of *S*. Typhimurium against complement lysis [29]. However, the results obtained in later experiments showed that it is the *wzz*_ST_ gene, which is essential for *S*. Typhimurium resistance to human serum [29, 34]. Bravo et al. showed that the survival of *S*. Enteritidis LPS length mutants in 40% NHS is directly linked with the growth phase. Mutation in the *wzz*_fepE_ gene was found to lead to serum resistance in the logarithmic growth phase but had no influence on the survival in the stationary phase [34].

C3 activation by the tested *S*. Enteritidis PCM 2817 strains in 50% NHS showed that mutants with a repertoire of longer O-specific chains on the bacterial cell surface (WT, Δ*wzz*_fepE_, Δ*wzz*_ST_) more effectively activated the C3 protein of the complement system (Fig. 5A). However, we observed differences in complement activation between the entire bacterial cells and isolated LPS molecules. The analysis of that activation by isolated LPS preparations showed that the Δ*wzz*_fepE_ LPS mutant activated the complement system to a higher degree than the LPS isolated from the WT strain (Fig. 5B). In contrast, C5b-9 deposition on entire bacterial cells was more abundant for the Δ*wzz*_ST_ strain than for the WT strain. This discrepancy might be related to the previously discussed method of LPS isolation (Fig. 6A) [56] or to the difference between the physical state of the LPS molecules on the intact bacterial surface vs. isolated LPS preparation immobilized on polystyrene. The observed discrepancy in the level of activation of the complement system for bacterial cells and isolated LPS molecules could mean that with a shorter O-antigen, there is lowered activation of the complement system. Based on flow cytometry experiments (Fig. 6) it can be concluded that the WT strain and Δ*wzz*_fepE_ mutant deposit the end products of the complement cascade on their surfaces, but the C5b-9 complex is released from the cell surface without lysing the bacterial cell (Fig. 6B). Similar conclusions were reached by Joiner et al. studying the mechanism of resistance of *S*. Minnesota smooth and rough strains exposed to human serum. The authors showed that in serum-resistant strains, complement components are deposited on the polysaccharide portion of LPS, which forms a physical barrier separating them from the lipid portion of the cell membrane [28, 64]. It would be useful to know the exact proportions between the individual O-antigen types (VL-OAg, L-OAg, LMW-OAg) of LPS in individual *S*. Enteritidis PCM 2817 mutants. If mutation in the Δ*wzz*_ST_ gene affects the activation of the complement system to a greater extent (Fig. 6A) than the mutation in the Δ*wzz*_fepE_ gene, it can be postulated that the appropriate density of LPS with long O-specific chains on the surface of the outer membrane is of key importance in protecting *S*. Enteritidis against complement lysis. Most likely, the higher density of L-OAg LPS on the Δ*wzz*_*f*epE_ mutant is protective against the incorporation of the C5b-9 complex into the bacterial cell membrane, while in the case of the Δ*wzz*_ST_ mutant, the density of VL-OAg LPS is probably not high enough to protect the bacteria from depositing C5b-9 on the outer membrane (Fig. 6). VL-OAg LPS, as shown in the experiments with monosaccharide markers of O-Ag and LPS core (Fig. 3A, B), constitute only a tiny molar part of the total LPS. This implies that VL-OAg molecules are very sparsely distributed on the bacterial surface. In that mutant the L-OAg LPS molecules are most probably replaced by LMW-OAg or LPS with one repeating unit, as the overall number of LPS molecules is constant (Fig. 3C). As it turns out from the SDS PAGE analysis, the molar content of VL-OAg LPS is low, and the lack of L-OAg in *wzz*_ST_ mutant is probably not compensated by VL-OAg LPS. The polysaccharide layer on the outer membrane is thus much more susceptible to the action of serum proteins. Therefore, despite prominent C5b-9 deposition, permeability data suggests that the forming MAC cannot penetrate the membrane (Fig. 6). L-OAg LPS in turn strongly activates the complement alternative pathway (Fig. 5B), which is apparently somehow attenuated by the presence of VL-OAg. In the presence of short and very short LPS molecules this activation capability is very low, although the perforation of the membrane built up of such short molecules is the strongest (Fig. 6B).

 In vivo experiments, using laboratory animals is an essential element in determining the degree of pathogenicity of bacterial strains and in understanding interactions between the host and the pathogen. Although the use of rodent models is widely used in the study concerning the pathogenesis of *Salmonella* infections, mammalian studies are often time-consuming, require an expensive experimental set up and are associated with significant ethical issues. The introduction of invertebrate models allows for a substantial reduction of research costs and minimizing the ethical concerns. Numerous studies have confirmed that the Wax moth *G. mellonella* is a convenient model to test some aspects of the pathogenicity of bacterial strains [65–71]. Bender et al. showed that the structure of LPS affects the virulence of *S*. Typhimurium NCTC 12023. *G. mellonella* survival after injection with mutant strains lacking the synthesis of VL-OAg or L-OAg was greater, than the survival of larvae after injection with the wild-type strain. In addition, the authors showed that removal of the entire O-specific antigen was associated with the loss of virulence of *S*. Typhimurium NCTC 12023 in the *G. mellonella* infection model [70]. Our investigations are in accordance with those observations, showing that the elimination of a particular fraction of O-antigen had an effect of the survival rate of *G. mellonella* larvae. After challenge with *S*. Enteritidis PCM 2817 WT and the Δ*wzz*_fepE_ most of the larval population did not survived (survival 13% and 7%, respectively) (Fig. 7A, CFU/ml 10^7^). The elimination of the L-OAg LPS reduced the pathogenicity to 27% of survival, indicating the importance of long O-antigens as a virulence factor of *S*. Enteritidis (Fig. 7A, CFU/ml 10^7^). The obtained results confirm the observations of Bender et al. [70] that the structure of LPS may affect the pathogenicity of strains in the infection model of *G. mellonella* larvae. An interesting observation is that the lack of VL-OAg increases bacterial virulence (increased larval mortality, Fig. 7A). Perhaps the bacteria produce other structures on the surface of the outer membrane that compensate for the lack of VL-OAg. Surprisingly, the survival rate of wax moth larvae subjected to infection with Δ*wzz*_fepE_ was lower than larvae infected with WT or double mutants. This suggests that VL-LPS can have a moderating role at least in invertebrates (Fig. 7A).

Previous research of other groups concentrated on immunological aspects of LPS length diversity, but the molecular characterization of the O-antigen length was limited to SDS-PAGE analysis with various types of LPS pattern visualization. In our work we have analyzed the length of LPS O-specific polysaccharide using mass spectrometry, therefore we could precisely compare the LPS modal distribution in particular mutants. The elaborated method utilizes tyvelose as an LPS length marker, which has advantages over the previously used method using sialic acid, which could not be used in strains lacking NeuAc [55]. Our method can be also used for quick *S*. Enteritidis strain identification by detection of Tyv. Previous publications by other groups have mainly studied the *S*. Typhimurium serotype, which is an predominant serovar in the United States. *S*. Enteritidis, which is prevalent in Europe and used in the present work, have not yet been studied in this regard. Several publication compare the immunological aspects of reactivity of different strains [28, 64] and different serotypes, where the O-antigen polysaccharide structure is not identical [63]. That approach may introduce variables into the experiment that will not be taken into account. In our publication, we use different mutants of the same *Salmonella* strain, differing only in the pattern of LPS modal distribution, which allows us to analyze the impact of just that single factor. We also analyze multiple aspects of complement activation, using not only survival in serum but also the more subtle aspects of complement system activation, like C3 activation, specific blocking of C5, C5b9 deposition, bacterial cell permeability and finally preliminary in vivo study on larvae model.

Although the phenomenon of modulation of the length of the lipopolysaccharide molecule probably cannot be used for vaccine construction, the different modal distribution of lipopolysaccharide and the absence of some LPS fractions, especially VL-OAg and L-OAg LPS, may lead to better exposure of some immunogenic structures, such as fimbriae or outer membrane proteins, on the bacterial surface. This in turn may make such bacteria more susceptible to attack by complement or specific antibodies, and vaccines based on the aforementioned structures may be more effective against these bacteria.

To summarize, the obtained data clearly demonstrate that *S*. Enteritidis bacteria require LPS with a specific modal length to resist complement-mediated lysis and to survive in the *G. mellonella* infection model. The results indicate a particular contribution of L-OAg in bacterial virulence. The observed inconsistency of the activation potential between isolated LPS and entire bacterial cells is intriguing and requires further study in future.

## Supplementary Information

Below is the link to the electronic supplementary material.

Supplementary file1 (DOCX 395 KB)

## Data Availability

No datasets were generated or analysed during the current study.
