# Supplementary material for: Lipopolysaccharide with long O-antigen is crucial for Salmonella Enteritidis to evade complement activity and to facilitate bacterial survival in vivo in the Galleria mellonella infection model
Source: Med Microbiol Immunol. 2024 May 20;213(1):8. doi: 10.1007/s00430-024-00790-3 (PMC11106168; doi:10.1007/s00430-024-00790-3)
Supplement: Supplementary file 1 — Supplementary file1 (DOCX 395 KB) [file 430_2024_790_MOESM1_ESM.docx]

4

5

6

7

8

9

10

11

12

13

14

15

0

5

10

15

20

25

30

35

40

45

50

55

60

65

70

75

80

85

90

95

100

9.94

10.12

10.22

10.31

7.15

10.70

11.42

12.30

11.79

12.59

12.92

12.11

13.33

7.98

13.58

7.68

7.39

4.93

9.75

5.02

11.20

5.73

15.08

9.37

13.90

5.53

8.51

6.43

9.33

14.51

6.63

4.78

8.32

6.14

4.10

Relative Abundance

Time (min)

Tyv

Rha

Kdo

Hep

MuAc

**Fig. S1** Chromatogram from the separation of sugar derivatives for bacterial cells of *S.* Enteritidis PCM 2817. Tyv – tyvelose; Rha – rhamnose; Hep – heptose; Kdo – 3-deoxy-D-*manno*-oct-2-ulosonic acid; MuAc – N-acetylmuramic acid.

A.

B.

C.

D.

E.

**Fig. S2** Mass spectra and structures of acetylated methylglycosides of Tyv (A); Rha (B); Hep (C); and derivatives of MuAc (D) and Kdo (E).


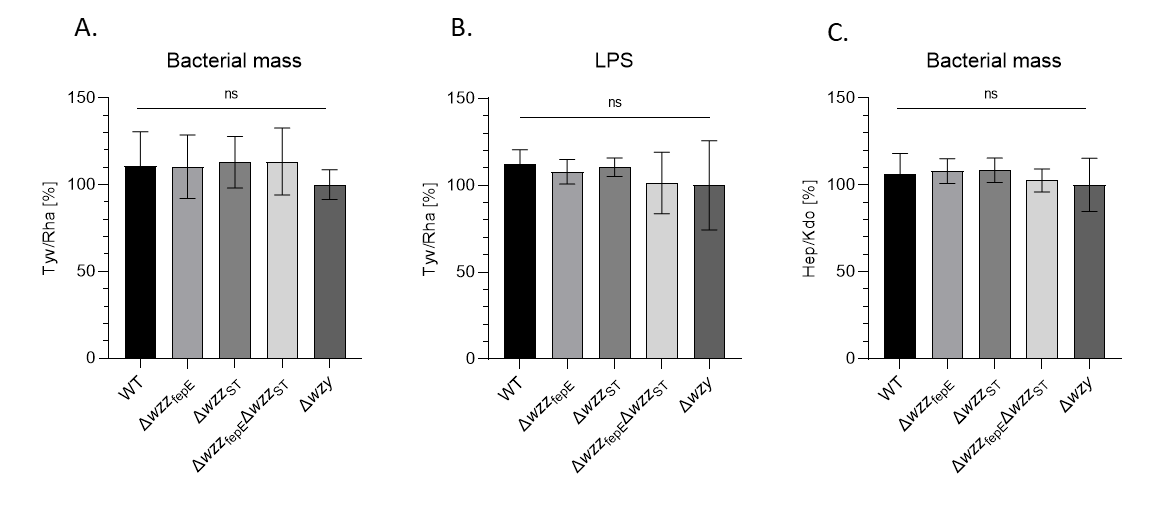


**Fig. S3** Comparison of the proportions of monosaccharides Tyv, Rha, Hep, Kdo in bacterial cells and isolated LPS preparations of *S*. Enteritidis PCM 2817 wild type and mutants. The result are presented as the ratio: Tyv/Rha for bacterial cells (A), Tyv/Rha for LPS (B), Hep/Kdo for bacterial cells (C), expressed as a percentage, taking the value of 100% for the Δ*wzy* mutant. The experiment was performed in six biological replicates. Results were analyzed with ordinary one-way ANOVA with Tukey's multiple comparisons test (*p<0.05, **p<0.01, ***p<0.001, ****p<0.0001). Average from six measurements ± SD.
